# Prognostic impact of pretherapeutic gamma-glutamyltransferase on patients with nasopharyngeal carcinoma

**DOI:** 10.1371/journal.pone.0172345

**Published:** 2017-02-27

**Authors:** Yue-Feng Wen, Xian-Zi Yang, Li-Si Zeng, Hai-Hua Peng, Wen-Jin Huang, Long-Mei Cai, Tong-Chong Zhou, Xiao-Dan Lin

**Affiliations:** 1 Department of Radiotherapy, Cancer Center of Guangzhou Medical University, Guangdong, China; 2 Guangzhou Institute of Oncology, Cancer Center of Guangzhou Medical University, Guangdong, China; University of South Alabama Mitchell Cancer Institute, UNITED STATES

## Abstract

**Background:**

Gamma-glutamyltransferase (GGT) is a membrane-bound enzyme involved in the metabolism of glutathione. Studies suggested that GGT played an important role in the tumor development, progression, invasion and drug resistance and prognosis. The association between GGT and prognosis of patients with nasopharyngeal carcinoma (NPC) was unknown. This study was conducted to investigate the association of pretherapeutic serum level of GGT with clinical-pathological parameters and survival in patients with NPC.

**Methods:**

Two hundred and twenty-two patients with NPC were recruited in this study and were stratified into two GGT risk groups (≤ 34.5 U/L, > 34.5 U/L). The association of pretherapeutic serum GGT levels with clinical–pathological parameters was examined. Univariate and multivariate survival analyses were performed.

**Findings:**

The pretherapeutic serum level of GGT was not associated with gender, age, pathology, T stage, N stage, TNM stage, chemotherapy or radiotherapy in patients with NPC. Patients in the high-risk GGT group had a poorer survival than the low-risk GGT group (3-year overall survival, 74.2% vs. 50.2%, P = 0.001; 3-year progression-free survival, 76.4% vs. 47.1%, P < 0.001; 3-year loco-regional relapse-free survival, 76.4% vs. 51.3%, P < 0.001; 3-year distant metastasis-free survival, 89.5% vs. 66.4%, P < 0.001). Multivariate analysis suggested that patients in the high-risk GGT group had 2.117 (95% confidence interval [CI], 1.225 ∼ 3.659, P = 0.007) times the risk of death, 2.836 (95% CI, 1.765 ∼ 4.557, P < 0.001) times the risk of progression, 2.551 (95% CI, 1.573 ∼ 4.138, P < 0.001) times the risk of relapse, and 3.331 (95% CI, 1.676 ∼ 6.622, P < 0.001) times the risk of metastasis compared with those in the low-risk GGT group.

**Conclusion:**

The pretherapeutic serum level of GGT might serve as a novel independent prognostic factor for overall-survival, progression-free survival, loco-regional relapse-free survival and distant metastasis-free survival in patients with NPC.

## Introduction

Nasopharyngeal carcinoma (NPC) is endemic in southern China and Southeast Asia, with high incidence rates of 20–30 cases per 100,000 population [1–3]. Chemoradiotherapy is the primary treatment modality for locoregionally advanced NPC. Although the TNM staging system is the most important prognostic indicator for NPC patients, patients with the same TNM stages and similar treatment regimens could have significantly different survival outcomes due to the tumor’s biological heterogeneity. In addition to the TNM staging system, more and more molecular biomarkers have been evaluated as potential prognosis predictors for NPC, including serum lactate dehydrogenase (LDH) [4], C-reactive protein (CRP) [5], D-dimer [6], fibrinogen [7], and plasma Epstein-Barr virus DNA (EBV DNA) [8]. Recently, pretreatment plasma EBV DNA levels have been increasingly employed for the diagnosis, risk stratification, monitoring, and prediction for the prognosis of NPC [8, 9].

Gamma-glutamyltransferase (GGT) is a cell-membrane bound enzyme involved in the metabolism of glutathione (GSH), catalyzing the degradation of extracellular GSH and subsequently promoting amino-acid recovery for intracellular GSH synthesis [10]. As GSH is the main water-soluble antioxidant within the cell, GGT has been recognized to contribute to cellular antioxidant defenses [10, 11]. Several previous studies revealed that GGT played a potentially important role in the tumor development, progression, invasion and drug resistance and prognosis [10, 12–16]. Elevated serum level of GGT was found to be associated with poorer prognosis in several human cancers, such as renal cell carcinoma [17], ovarian cancer [18], esophageal squamous cell carcinoma [19], breast cancer [20], endometrial cancer [21], as well as cervical cancer [22].

To the best of our knowledge, there have been few report about the prognostic impact of pretherapeutic serum level of GGT on patients with NPC in detail untill now. A recent study aimed to investigate the association of serum LDH and ALP with NPC showed that increased GGT level (> 50 U/L) had no significant impact on survival of patients with NPC. However, it also indicated that patients with higher GGT level had a worse survival when by using the optimal cutoff value of GGT (28.5 U/L) determined by receiver operative characteristic (ROC) curve [23]. It seemed that different cut-off values led to different conclusions. Therefore, we performed this study to further investigate the association between pretherapeutic serum level of GGT and the clinical-pathological parameters and prognosis in the patients with NPC.

## Materials and methods

### Patients

A total of 222 patients with primary NPC were consecutively recruited from January 2011 to December 2014 at the Cancer Center of Guangzhou Medical University, China. This study was reviewed and approved by the institutional review board and ethics committee of Cancer Center of Guangzhou Medical University. Written informed consent was obtained from all patients. Patients who presented with pre-existing comorbidities, known to be related with elevation of GGT (i.e. hepatobiliary tract, pancreatic and heart disease or alcohol abuse) were excluded from this study (number = 128).

### Clinical management

The pre-treatment evaluation included a complete patient history, physical examinations, haematology and biochemistry profiles, fibreoptic nasopharyngoscopy, chest X-ray, abdominal sonography, magnetic resonance imaging (MRI) of the nasopharynx and neck, and whole-body bone scan or whole-body FDG PET/CT. All patients were restaged according to the seventh American Joint Committee on Cancer (AJCC) TNM staging manual. In total, 21 (9.5%) patients were treated with three-dimensional conformal radiotherapy radiotherapy (3DCRT), and 201 (90.5%) patients were treated with intensity-modulated radiotherapy (IMRT). In addition, 209 (94.1%) patients with stage II–IV disease received platinum-based chemotherapy. A stratified multitherapeutic protocol was used. Radiation alone was administered for stage I disease, and radiation alone or with concurrent platinum-based chemotherapy was administered for stage II disease [24]. Concurrent chemoradiotherapy with or without neoadjuvant or adjuvant chemotherapy was administered for advanced-stage disease (stages III and IV). Neoadjuvant or adjuvant chemotherapy consisting of cisplatin plus 5-fluorouracil or cisplatin plus taxane was administered every 3 weeks for two or three cycles [25]. Concurrent cisplatin chemotherapy was administered every 3 weeks. All patients were treated according to the principles of treatment for NPC patients at the Cancer Center of Guangzhou Medical University.

### GGT measurement

Blood samples for the evaluation of serum GGT levels were obtained by peripheral venous puncture 24–48 h prior to therapy. Gamma-glutamyltransferase was routinely determined to rule out liver damage before treatment starts. Gammaglutamyltransferase concentrations were analysed with an enzyme kinetic assay (Modular Hitachi 7600 and Hitachi 7080, Hitachi High-Technologies Corporation Tokyo, Japan).

### Statistical analysis

Values were described by mean (standard deviation [SD]) when normally distributed or by median when presented with skewed distribution. The Mann–Whitney *U* test and chi-square test were performed to analyse the association between pretherapeutic serum level of GGT and clinical-pathological parameters. Receiver operative characteristic (ROC) curve was used to determine the optimal cutoff value of GGT for survival. According to the cutoff value, serum level of GGT was divided into two groups, high-risk group and low-risk group. Survival probabilities were calculated by the Kaplan–Meier method. Differences between groups were measured using the log-rank test. Survival times of patients still alive or dead as a result of other causes than cancer were censored with the last follow-up date. The primary end point of this study was progression-free survival (PFS). PFS was defined as the duration from the date of definite diagnosis to the date of disease progression or censored at the date of last follow-up. The secondary end points include overall survival (OS), locoregional relapse-free survival (LRRFS), distant metastasis-free survival (DMFS). OS was calculated from the time of definite diagnosis to the time of death from any cause or to the time of last follow-up (at which time data were censored). LRRFS and DMFS were also evaluated and calculated from the date of definite diagnosis until the day of first locoregional or distant relapse or until the date of the last follow-up visit. Univariate and multivariate analysis was performed using Cox’s proportional hazards regression model with a forward stepwise procedure (the entry and removal probabilities were 0.05 and 0.10, respectively). Analyses were performed using the statistical software package SPSS 20.0 (SPSS, Chicago, IL) and Graph Pad Prism for windows, version 6 (Graph Pad Prism, San Diego, CA, USA). A two-sided *P*-value less than 0.05 was considered statistically significant.

## Results

### Patients’ characteristics

**Fig 1:**
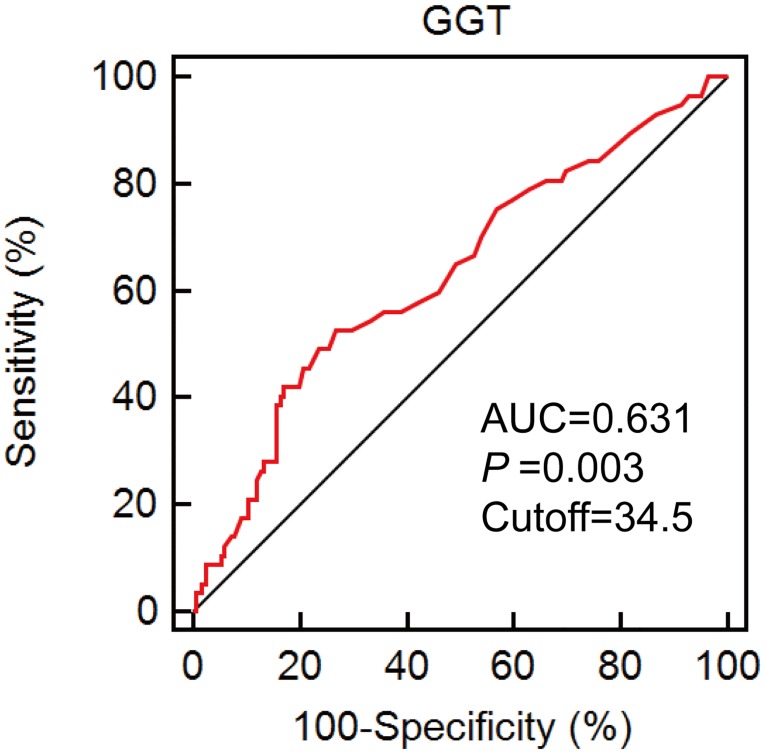
ROC curve using pretherapeutic serum level of GGT. The optimal cut-off value was 34.5, with a sensitivity of 51.7% and a specificity of 73.2%.

A total of 222 patients with primary NPC were included in the final analysis. The clinical–pathological characteristics of the study cohort were presented in Table 1. The optimal cutoff value of serum level of GGT with the best discriminatory power was determined to be 34.5 U/L according to ROC curve analysis (Fig 1). A total of 148 patients were assigned to the low-risk group (GGT ≤ 34.5 U/L) and 74 patients were assigned to the high-risk group (GGT > 34.5 U/L).

**Table 1:** Association of pretherapeutic serum level of GGT and clinical–pathological characteristics in patients with NPC.

Characteristics	Patients N (%)	GGT (U/L)	P-value*	GGT		P-value**
		Median (Mean)		Low-risk group (%)	High-risk group (%)	
Gender						
Male	164 (73.9)	28.0 (38.0)	0.79	111 (67.7%)	53 (32.3%)	0.59
Female	58 (26.1)	18.5 (39.3)		37 (63.8%)	21 (36.2%)	
Age (years)						
≤ 45	98 (44.1)	26.5 (38.9)	0.81	60 (61.2%)	38 (38.8%)	0.127
> 45	124 (55.9)	28.0 (37.9)		88 (71.0%)	36 (29.0%)	
Pathology (WHO type)						
I	1 (0.4)	19.0 (19.0)	0.45	1 (100%)	0 (0.0%)	0.299
II	116 (52.3)	26.5 (34.9)		82 (70.7%)	34 (29.3%)	
III	105 (47.3)	28.0 (42.3)		65 (61.9%)	38 (38.1%)	
T stage						
T1	8 (3.6)	30.5 (39.9)	0.21	5 (62.5%)	3 (37.5%)	0.262
T2	85 (38.3)	27.0 (32.7)		61 (71.8%)	24 (28.2%)	
T3	77 (34.7)	27.0 (38.7)		53 (68.8%)	24 (31.2%)	
T4	52 (23.4)	30.5 (46.6)		29 (55.8%)	23 (44.2%)	
N stage						
N0	16 (7.2)	23.5 (38.9)	0.16	12 (75.0%)	4 (25.0%)	0.236
N1	74 (33.3)	25.5 (34.3)		53 (71.6%)	21 (28.4%)	
N2	103 (46.4)	28.0 (36.7)		68 (66.0%)	35 (34.0%)	
N3	29 (13.1)	33.0 (53.9)		15 (51.7%)	14 (48.3%)	
TNM stage						
I	3 (1.4)	24.0 (28.0)	0.08	2 (66.7%)	1 (33.3%)	0.074
II	40 (18.0)	26.5 (34.0)		29 (72.5%)	11 (27.5%)	
III	106 (47.7)	26.5 (34.2)		77 (72.6%)	29 (27.4%)	
IV	73 (32.9)	32.0 (47.0)		40 (54.8%)	33 (45.2%)	
Chemotherapy						
Radiotherapy alone	13 (5.9)	34.0 (40.8)	0.78	8 (61.5%)	5 (38.5%)	0.687
Chemoradiotherapy	209 (94.1)	27.0 (38.2)		140 (67.0%)	69 (33.0%)	
Radiotherapy						
3DCRT	21 (9.5)	32.0 (32.4)	0.15	13 (61.9%)	8 (38.1%)	0.627
IMRT	201 (90.5)	27.0 (38.9)		135 (67.2%)	66 (32.8%)	

NOTE:

*Kruskal–Wallis test.

**Chi-square test.

TNM, tumor node metastasis.

### Association between pretherapeutic serum level of GGT and clinical–pathological characteristics

The pretherapeutic serum level of GGT was not associated with gender, age, pathology, T stage, N stage, TNM stage, chemotherapy or radiotherapy. No association between GGT and clinical–pathological characteristics was found when analying the categorical variables by chi-square test either (Table 1).

### Association between pretherapeutic serum level of GGT and prognosis

**Fig 2:**
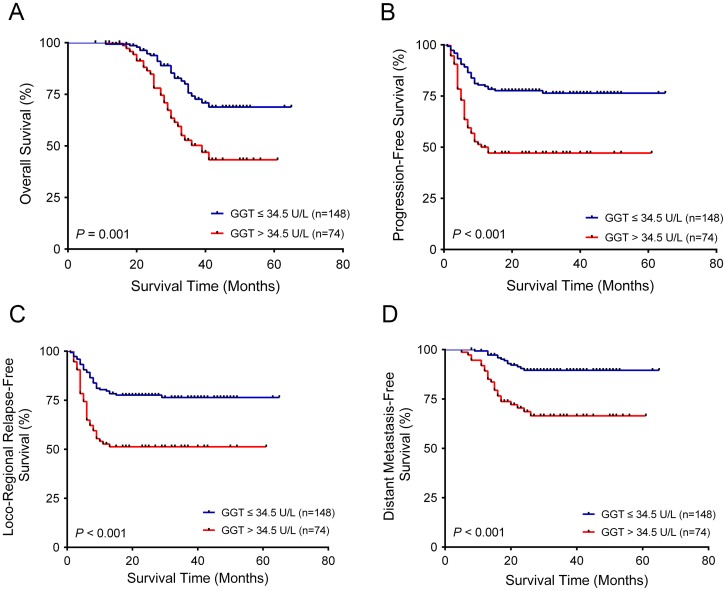
Kaplan–Meier curves for OS, PFS, LRRFS and DMFS regarding the pretherapeutic serum level of GGT. Kaplan-Meier survival estimates and log-rank tests were used to analyze the prognostic significance of GGT in all patients. (a) OS; (b) PFS; (c) LRRFS; (d) DMFS.

In univariate survival analysis, high-risk level of GGT, advanced T stage, advanced N stage, and advanced TNM stage were associated with poorer OS, PFS, LRRFS and DMFS (Tables 2 and 3). The 3-year OS, PFS, LRRFS and DMFS rates for patients in the low-risk GGT group and the high-risk GGT group were 74.2% vs. 50.2% (P = 0.001), 76.4% vs. 47.1% (P < 0.001), 76.4% vs. 51.3% (P < 0.001), and 89.5% vs. 66.4% (P < 0.001), respectively. Kaplan–Meier curves were shown in Fig 2 for the two groups. Multivariate analysis suggested that patients in the high-risk GGT group had 2.117 (95% confidence interval [CI], 1.225 ~ 3.659, P = 0.007) times the risk of death, 2.836 (95% CI, 1.765 ~ 4.557, P < 0.001) times the risk of progression, 2.551 (95% CI, 1.573 ~ 4.138, P < 0.001) times the risk of relapse, and 3.331 (95% CI, 1.676 ~ 6.622, P < 0.001) times the risk of metastasis compared with those in the low-risk GGT group (Tables 2 and 3). Furthermore, advanced T stage was also associated with poorer OS, PFS, LRRFS and DMFS in multivariable survival analyses (Tables 2 and 3).

**Table 2:** Univariate and multivariate analysis of pretherapeutic serum level of GGT associated with OS and PFS in patients with NPC.

Variables	Univariate analysis		P value	Multivariate analysis		P value
	HR	95% CI		HR	95% CI	
**Overall Survival**						
Gender (Female vs. Male)	0.539	0.264–1.099	0.089	0.709	0.339–1.484	0.362
Age (> 45y vs. ≤45y)	0.950	0.565–1.599	0.848	0.722	0.421–1.236	0.234
Pathology (III vs. I-II)	0.920	0.547–1.547	0.753	0.597	0.345–1.034	0.066
T stage (T3-4 vs. T1-2)	3.612	1.870–6.978	< 0.001	2.927	1.219–7.029	0.016
N stage (N2-3 vs. N0-1)	2.916	1.607–5.291	< 0.001	2.205	1.046–4.646	0.038
TNM stage (III-IV vs. I-II)	4.004	1.595–10.049	0.003	0.877	0.215–3.568	0.854
Radiotherapy (IMRT vs. 3D-CRT)	7.850	1.086–56.739	0.041	7.502	1.009–55.762	0.049
GGT expression (high vs. low)	2.418	1.437–4.069	0.001	2.117	1.225–3.659	0.007
**Progression-Free Survival**						
Gender (Female vs Male)	0.562	0.308–1.023	0.059	0.548	0.298–1.009	0.053
Age (> 45y vs. ≤45y)	0.964	0.608–1.529	0.877	0.882	0.549–1.418	0.605
Pathology (III vs. I-II)	1.002	0.633–1.585	0.995	0.773	0.481–1.242	0.287
T stage (T3-4 vs. T1-2)	3.187	1.829–5.553	< 0.001	3.17	1.578–6.368	0.001
N stage (N2-3 vs. N0-1)	1.805	1.094–2.978	0.021	1.594	0.886–2.871	0.12
TNM stage (III-IV vs. I-II)	3.156	1.368–7.281	0.007	0.896	0.277–2.897	0.854
Radiotherapy (IMRT vs. 3D-CRT)	2.877	0.906–9.140	0.073	3.328	1.030–10.752	0.045
GGT expression (high vs. low)	2.865	1.806–4.546	< 0.001	2.836	1.765–4.557	< 0.001

NOTE: TNM, tumour node metastasis. HR, hazard ratio; CI, confidence interval.

**Table 3:** Univariate and multivariate analysis of pretherapeutic serum level of GGT associated with LRRFS and DMFS in patients with NPC.

Variables	Univariate analysis		*P* value	Multivariate analysis		*P* value
	HR	95% CI		HR	95% CI	
**Loco-regional Relapse-Free Survival**						
Gender (Female vs. Male)	0.475	0.250–0.905	0.024	0.438	0.228–0.843	0.013
Age (> 45y vs. ≤45y)	1.074	0.669–1.724	0.768	0.987	0.606–1.605	0.957
Pathology (III vs. I-II)	1.036	0.648–1.655	0.884	0.854	0.528–1.383	0.521
T stage (T3-4 vs. T1-2)	3.238	1.828–5.735	< 0.001	3.310	1.593–6.875	0.001
N stage (N2-3 vs. N0-1)	1.681	1.014–2.786	0.044	1.461	0.808–2.640	0.209
TNM stage (III-IV vs. I-II)	2.989	1.293–6.906	0.010	0.837	0.254–2.763	0.771
Radiotherapy (IMRT vs. 3D-CRT)	2.731	0.859–8.685	0.089	3.000	0.930–9.677	0.066
GGT expression (high vs. low)	2.580	1.612–4.128	< 0.001	2.551	1.573–4.138	< 0.001
**Distant Metastasis-Free Survival**						
Gender (Female vs. Male)	0.549	0.229–1.317	0.179	0.590	0.243–1.428	0.242
Age (> 45y vs. ≤45y)	0.588	0.307–1.127	0.109	0.525	0.268–1.031	0.061
Pathology (III vs. I-II)	1.163	0.610–2.216	0.647	0.761	0.388–1.490	0.425
T stage (T3-4 vs. T1-2)	4.285	1.786–10.280	0.001	3.063	1.165–8.051	0.023
N stage (N2-3 vs. N0-1)	4.048	1.688–9.708	0.002	2.914	1.107–7.676	0.030
TNM stage (III-IV vs. I-II)	10.240	1.403–74.734	0.022	1.940	0.183–20.547	0.582
Radiotherapy (IMRT vs. 3D-CRT)	4.253	0.583–31.028	0.153	4.949	0.656–37.33	0.121
GGT expression (high vs. low)	3.876	1.993–7.536	< 0.001	3.331	1.676–6.622	0.001

NOTE: TNM, tumour node metastasis. HR, hazard ratio; CI, confidence interval.

## Discussion

In the present study, we investigated the associations of the serum level of pretherapeutic GGT with the clinical–pathological parameters and prognosis of NPC. To the best of our knowledge, there have been few report on the prognostic impact of pretherapeutic serum level of GGT on patients with NPC in detail up to now. We stratified the patients into high-risk group and low-risk group, according to the best cutoff value determined by ROC curve. We demonstrated that high-risk group of GGT was associated with poorer prognosis in patients with NPC. Pretherapeutic serum level of GGT was an independent prognostic factor for patients with NPC. Patients in the high-risk GGT group had significant worse 5-year OS, PFS, LRRFS and DMFS than patients in the low-risk GGT group. Patients in the high-risk GGT group had higher risks of death, progression, relapse and metastasis than those in the low-risk GGT group. Our finding was similar to a recent study which showed that NPC patients with higher GGT level (> 28.5 U/L) had a worse survival except that the cut-off value was different [23]. Furthermore, our finding was also consistent with findings of the previous studies on prognostic relevance of pretherapeutic serum level of GGT in several other cancers, such as esophageal squamous cell carcinoma [19, 26], cervical cancer [22], ovarian cancer [18], renal cell carcinoma [17], and endometrial cancer [21]. Higher level of GGT was indicated to be associated with poorer prognosis in cervical cancer [22].

Although previous studies have indicated that GGT might play a meaningful role in tumor cell biology, the exact functional mechanisms remain unclear. Several potential mechanisms through which GGT impacts cancer biology have been postulated. GGT was demonstrated to participate in the important redox-sensitive processes, such as antioxidant/antitoxic defenses and cellular proliferative/apoptotic balance [27, 28], thereby function as an antioxidative role, as well as a prooxidative role within the tumour microenvironment. On the one hand, GGT was found to play a crucial role in the metabolism of glutathione which was the major thiol antioxidant in the body, consequently protecting cells against further oxidative stress [10, 29, 30]. GGT could generate an additional source of reactive oxygen species (ROS) during glutathione metabolism, which was implicated to modulate a series of biological reactions involving cellular growth, proliferation and apoptosis [14, 28, 31], and the ROS-related genes redox regulation seemed to modulate GGT expression in reflect [12]. Therefore, the continuous production of ROS generated by increased GGT expression in tumor cells may contribute to tumor progression and invasion. Moreover, GGT was indicated to be upregulated after oxidative stress through the Ras–mitogen-activated protein kinase (MAPK) pathways in rat colon carcinoma cell [32].

On the other hand, GGT and GSH were regarded as essential components of the antioxidant defence by quenching free radicals on DNA [10, 11]. The previous study revealed that GGT act as pro-oxidant functions, impairing cellular proliferative/apoptotic balance, sequently modulating tumour formation and progression [12]. GSH was indicated to mediate the reduction of phosphatase and tensin homolog (PTEN), which act as a tumour suppressor by inhibiting phosphoinositide 3-kinasedependent activation of AKT [33].

Furthermore, evidences indicated that GGT mRNA might be induced by several cytokines, such as tumor necrosis factor alpha (TNF-alpha) [34], and interferon (IFN)-alpha and–beta [35]. TNF-alpha was implicated to induce GGT expression via nuclear factor-kappaB (NF-κB)-dependent signaling, in cooperation with specificity protein 1 (Sp1) transcription factor and RNA polymerase II recruitment to the GGT promoter [36]. The results above perhaps imply that GGT may participate in the inflammation processes mediated by specific inflammatory cytokines [12].

As a retrospective study, our study was limited by biases such as lack of random assignment, and patient’s incomplete data acquisition. Nonetheless, patients with clinically relevant comorbidities known to be associated with elevated GGT, such as hepatobiliary tract, pancreatic and heart disease or alcohol abuse were excluded from the study. Despite the potential limitations, our results were clinically valuable.

We conclude that pretherapeutic serum level of GGT might be a novel independent prognostic factor for OS, PFS, LRRFS and DMFS in patients with NPC. Patients with higher level of GGT have poorer prognosis. However, whether GGT itself has a direct etiological role in carcinogenesis or may just be a marker of an underlying etiology needs further research.

## Supporting information

S1 Dataset The origin dataset of the manuscript. (XLSX)
